# Systematic screening of gas diffusion layers for high performance CO_2_ electrolysis

**DOI:** 10.1038/s42004-023-00836-2

**Published:** 2023-02-24

**Authors:** Angelika Anita Samu, Imre Szenti, Ákos Kukovecz, Balázs Endrődi, Csaba Janáky

**Affiliations:** 1 Department of Physical Chemistry and Materials Science, University of Szeged, Rerrich Square 1, Szeged, H-6720 Hungary; 2 eChemicles Zrt, Alsó Kikötő sor 11, Szeged, H-6726 Hungary; 3 Department of Applied and Environmental Chemistry, University of Szeged, Rerrich Square 1, Szeged, H-6720 Hungary

**Keywords:** Electrocatalysis, Carbon capture and storage, Electrocatalysis

## Abstract

Certain industrially relevant performance metrics of CO_2_ electrolyzers have already been approached in recent years. The energy efficiency of CO_2_ electrolyzers, however, is yet to be improved, and the reasons behind performance fading must be uncovered. The performance of the electrolyzer cells is strongly affected by their components, among which the gas diffusion electrode is one of the most critical elements. To understand which parameters of the gas diffusion layers (GDLs) affect the cell performance the most, we compared commercially available GDLs in the electrochemical reduction of CO_2_ to CO, under identical, fully controlled experimental conditions. By systematically screening the most frequently used GDLs and their counterparts differing in only one parameter, we tested the influence of the microporous layer, the polytetrafluoroethylene content, the thickness, and the orientation of the carbon fibers of the GDLs. The electrochemical results were correlated to different physical/chemical parameters of the GDLs, such as their hydrophobicity and surface cracking.

## Abstract (web summary)

For economic viability, CO_2_ electrolyzers must meet a range of criteria such as low cell voltage, high current density, high product selectivity and long-term stability—which continues to be a challenge. Here, the authors systematically screen which parameters of twenty commercially available gas diffusion layers affect cell performance the most.

## Introduction

One of the most urgent challenges of our society is the high and continuously rising level of CO_2_ in the atmosphere, caused by anthropogenic emissions^1,2^. Chemical capture (e.g., amine absorption^3^) of CO_2_ from point sources, together with storage technologies^4^ (e.g., geological or in deep sea) are increasingly used strategies to decrease emissions. The electrochemical reduction of CO_2_ (CO_2_ reduction reaction, CO_2_RR) is an alternative route to neutralize harmful CO_2_ and convert it into useful chemicals and fuels (e.g., carbon monoxide, methane, ethylene)^5^ in the same process. A further benefit of the electrochemical CO_2_RR is that the energy requirement can be directly supplied from renewable energy sources (e.g., solar or wind energy)^6–8^.

To become an economically viable industrial technology^9^, CO_2_ electrolyzers must meet several strict criteria:^10,11^ operate at (i) low cell voltage, (ii) high current density, (iii) with high product selectivity and (iv) for a long time, without any notable change in the former parameters (i.e., durability). In the standard H-cell configuration using aqueous electrolytes, the availability of CO_2_ near the electrode surface is limited by its solubility, resulting in a large diffusion layer thickness, and therefore limited reaction rate^12,13^. A viable approach to overcome this problem is to provide CO_2_ directly as gas to the cathode^14^. This way, the diffusion layer thickness can be decreased by several orders of magnitude^5,13^. Gas diffusion electrodes (GDEs) are used in such electrochemical cells, in which the goal is to ensure the formation of a large triple phase boundary (or a situation close to this, with a very narrow diffusion layer in liquid phase) among the reactant gas (CO_2_), the catalyst nanoparticles, and the electrolyte solution^15–17^.

Such GDEs encompass the catalyst layer, immobilized on a gas diffusion layer (GDL)^18^. Depending on the employed catalyst, the CO_2_RR results in different products^19,20^. The reaction rate and the selectivity are also strongly affected by the structure of the electrolyzer cell and its components, which together with the operational conditions, define the microenvironment of the catalyst^21,22^. Here we focus on the cathode GDL, which allows the gas phase reactant, CO_2_, to reach the catalyst layer. One of the most important functions of the GDL is to provide a high and homogeneous surface area to the catalyst layer, hence improving the electrical contact and the cell performance. Beyond this, the GDL must be electrically conductive and mechanically robust. It is responsible for the transport of CO_2_ gas to the catalyst layer, and also participates in the water management, aiding to remove excess water from the catalyst surface. This means that beyond its composition, the morphology is also of prime importance.

The most frequently studied GDLs are carbon-based structures. The application of carbon materials has many advantages, such as their natural abundance, customizable porous structure, high (and tunable) surface area, good electrical conductivity, high-temperature stability, environmental friendliness, and affordable price^23^. Different types of carbon-based GDLs were previously studied in different applications ranging from oxygen depolarized chlor-alkali cathodes^24,25^ to fuel cell electrodes^26,27^. However, only a few studies focus on the behavior of different GDLs in the CO_2_RR, and to the best of our knowledge, no thorough investigation has been reported so far. Some GDLs are more frequently used than others, but our impression is that most researchers choose carbon papers for CO_2_RR that were proven to be suitable in other research fields, such as in PEM/AEM H_2_-O_2_ fuel cells. The conditions in CO_2_ electrolyzer cells, however, are notably different in terms of process temperature, the presence of physical water at the anode and CO_2_ and carbonate/bicarbonate ions at the cathode; hence conclusions cannot be directly translated from one field to another.

There are many types of commercially available carbon-based support layers (e.g., carbon cloth, felt, mesh, foam, and paper), which can have different effects on the CO_2_RR. Typically, the GDLs consist of a carbon fiber layer (CFL, often called as macroporous layer) and, in most cases, a microporous layer (MPL) immobilized thereon. There are two main groups of CFLs, woven and non-woven type. The carbon cloth group consists of intertwined, woven CFLs, while carbon papers are non-woven^28^. The structure of the non-woven CFLs can be further divided into two types: straight, ordered and randomly oriented (spaghetti-like) structures. The main effect of the MPL, which typically consist of carbon nanofibers or compressed carbon powder, is to ensure homogeneous catalyst-, gas- and current distribution, and to determine the roughness of the surface^24^. It is worth noting that some important properties, such as the gas permeation and the electrical resistance of these GDLs, are highly dependent on the measurement conditions and the experimental setup. These parameters vary notably upon compressing the GDL. As the optimal compression ratio might depend on the thickness and structure of the GDL, fair comparison among different GDLs can only be achieved if the compression ratio is optimized on a case-by-case basis.

The composition of the GDL can be further tailored to increase hydrophobicity, and therefore extend the durability of the electrochemical cell^29^. Both the CFL and the MPL can contain hydrophobing and binder agents, such as the most frequently employed polytetrafluoroethylene (PTFE). The effect of the PTFE loading in the GDL has already been studied, revealing a trade-off between the selectivity increase towards the CO_2_ reduction products formation (and the parallel suppression of HER) caused by the increasing hydrophobicity, and the increasingly hindered gas transport properties and decreasing electrical conductivity^30,31^. The presence of an MPL, the porosity and crack formation on the GDL (or GDE) all have a great influence on the electrolyte management, hence affecting the rate and selectivity of CO_2_RR^32–35^.

The effects of the physical parameters of the GDL (thickness, CFL, MPL) and PTFE content of the CFL and/or MPL on the mass transport of the reactants into and out of the catalyst layer have also been studied^36^. The general conclusion from these scarce studies was that the wetting properties of different GDLs affect the flooding of the GDEs during CO_2_RR, that is the accumulation of water in the GDE, leading to decreased selectivity and/or cell performance^22,37^. Finally, the GDL structure greatly affects the salt precipitation in the cathode GDE^38^, and consequently the stable and selective operation of the electrolyzer cells.

Our aim in this contribution was to provide a comprehensive assessment of the most frequently used commercially available GDLs (20 in total) in CO_2_RR. To allow a meaningful comparison of different GDLs, we studied the effect of the MPL, the PTFE content, the thickness, and the structure of the selected GDLs. Our goals include to highlight some general conclusions that can help in the future the rational design of GDLs in this field.

## Results and discussion

**Fig. 1:**
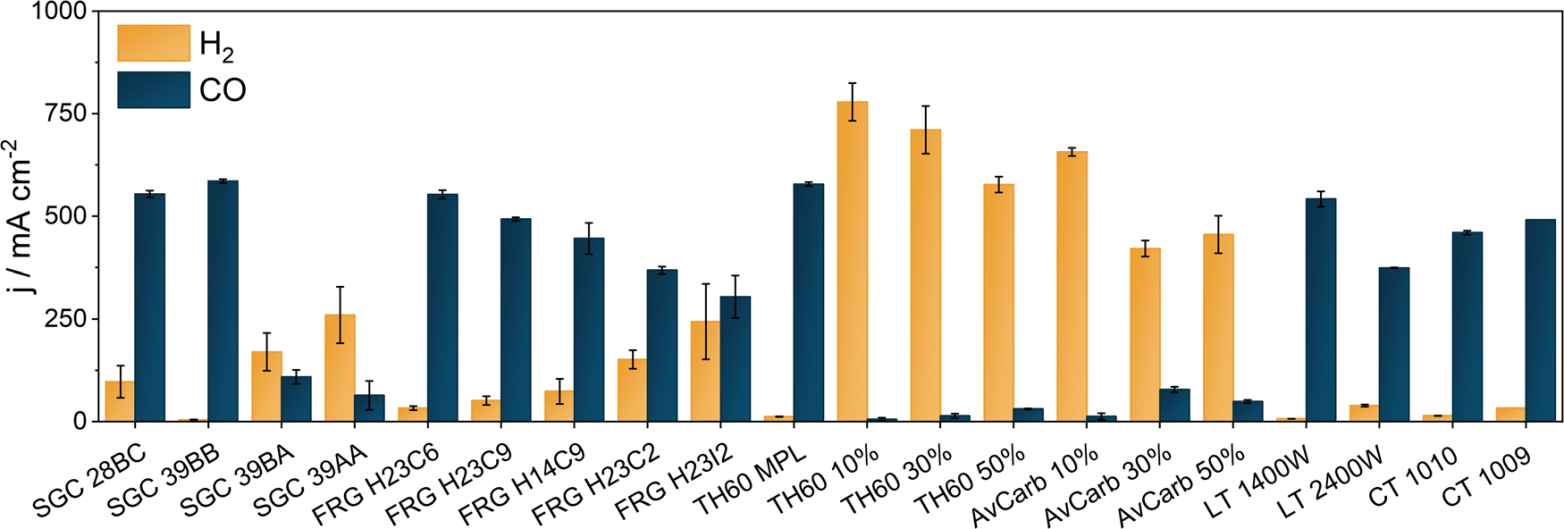
Comparing commercially available carbon gas diffusion layers-based gas diffusion electrodes in the CO_2_ reduction reaction. Partial current densities for CO (blue) and H_2_ (yellow) production during 1-hour long chronoamperometric CO_2_ reduction reaction measurements at ΔU = 3.0 V, applying different GDL based cathode GDEs with Ag catalyst. The electrolyte solution was 0.1 M CsOH, humidified CO_2_ was fed to the cathode at a flow rate of 12.5 cm^3^ cm^−2^ min^−1^ and the temperature of the electrolyzer cell was 60 °C. The error bars represent the deviations of two consecutive analyses during the same measurements.

We tested the operation of the same zero-gap electrolyzer cell in the CO_2_RR using 20 different commercially available GDLs from six manufacturers, under identical experimental conditions (see the Experimental Section later). To avoid any uncertainty related to catalyst synthesis, commercially available silver nanoparticles were used to form GDEs. These were spray-coated on the GDLs to form a catalyst layer (thickness ca. 25 µm, see details in the Supporting Information). The 1-hour long chronoamperometric measurements (see Supplementary Fig. 1 for the measurement sequence protocol), performed consecutively at three different cell voltages (Fig. 1 and Supplementary Figs. 2–3) revealed some general trends and important differences. By increasing the cell voltage, we achieved higher overall current densities for all GDLs, but at higher voltages the HER also became favorable, while the CO_2_RR selectivity decreased. During the electrolysis, only CO and H_2_ products were detected, with a total Faradaic Efficiency (FE) close to 100%. Importantly, in case of five of the tested GDLs (SGC 28BC, SGC 39BB, FRG H23C6, TH60 MPL and LT 1400 W), the CO partial current density exceeded 500 mA cm^−2^ already at 3.0 V (Fig. 1). The maximum CO partial current density was almost 600 mA cm^−2^ at 3.0 V, using the SGC 39BB GDL.

**Fig. 2:**
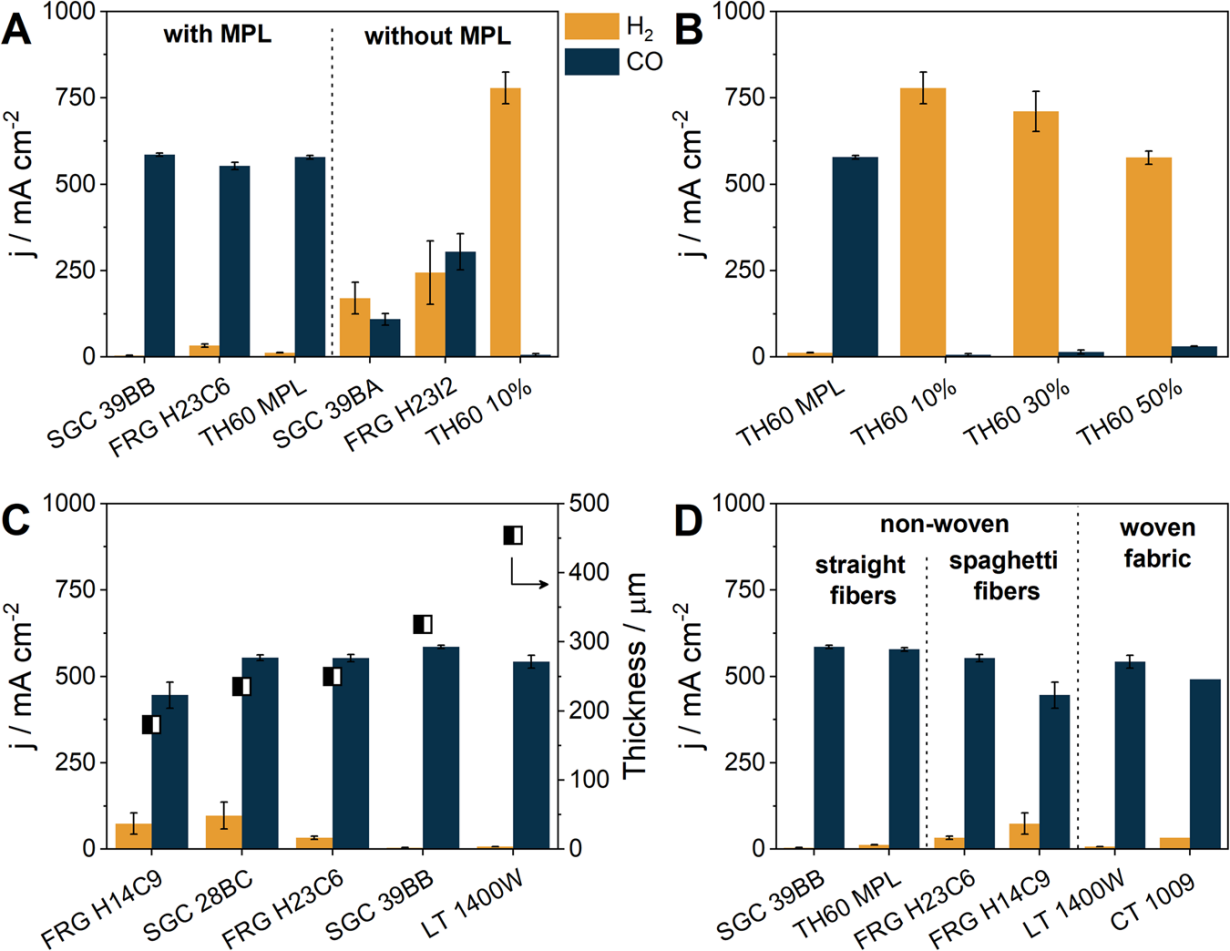
The effect of GDL structural parameters on the rate and selectivity of CO_2_ reduction reaction. The results from Fig. 1., categorized to demonstrate the effect of the (**A**) microporous layer (MPL), the (**B**) PTFE content, the (**C**) thickness (half black-half white squares), and the (**D**) structure of the gas diffusion layer. The error bars represent the deviations of two consecutive analyses during the same measurement.

To unravel which structural parameters affect the electrolyzer performance the most, the results were compared along a few selected properties: (i) whether they contained MPL or not, (ii) the PTFE content, (iii) the overall thickness, and (iv) the structure of the GDL (Fig. 2A-D, accordingly). A striking effect is seen when comparing GDLs that are based on the same CFL with and without MPL (Fig. 2A). GDLs from three manufacturers were selected (SGC, FRG, TH60), and the MPL-containing and MPL-free pairs were compared. For all three brands, a low CO_2_RR selectivity was found without MPL, while CO formed with above 90% FE with the MPL containing counterparts. We think that the addition of MPL to the GDL contributes to the higher CO_2_RR selectivity via at least two effects: first, by its hydrophobicity due to its PTFE content^35^, second, by increasing the contact area between the reactant and the catalyst due to its homogeneous, high surface area. We aimed to determine the electrochemically active surface area of these GDLs by cyclic voltammetry in aqueous solutions, as this can notably affect the process efficiency. These experiments resulted in unrealistically low values due to the hydrophobicity of the MPL. Therefore, we concluded that the most realistic comparison of these GDLs is provided by measuring the current density and product selectivity during operation conditions, as it is presented here.

The hydrophobicity of the GDE is clearly a critical parameter, as indicated by several earlier studies^11,17,36^. To investigate whether a highly hydrophobic CFL can be sufficient for selective CO_2_RR, we studied the effect of the PTFE content on the CO_2_RR in the Toray carbon papers subgroup (Fig. 2B). As gleaned from these results, even if the CFL contained a large amount of PTFE, it had no significant effect on the process selectivity in the absence of the MPL. Only a minor decrease in the overall current density can be observed with the increasing PTFE content. Based on the electrochemical impedance spectroscopy (EIS) measurements, the current decrease is not rooted in any electrical resistance increase, as it was found to be unaffected by the PTFE content (Supplementary Fig. 4). However, if the CFL contained only 8% PTFE, but there was also a MPL layer, the CO partial current density exceeded 500 mA cm^−2^, showing—again—that the existence of a MPL is necessary for efficient CO_2_RR.

The impact of the GDL thickness was not substantial (Fig. 2C). Interestingly, the thinnest was not the best, although intuitively a small thickness and hence small electrical resistance would be expected to lead to the best results. In fact, the electrical resistance of the cell—determined from EIS measurements—was found to be the same within experimental error for all samples in this comparison. This was also confirmed by calculations showing that the high-frequency resistance of the cell is mainly rooted in the membrane resistance (see the discussion after Supplementary Fig. 4).

The CO selectivity was much better for the thicker GDLs; the CO/H_2_ ratio was 6 for SGC 28BC, and 146 for the thicker SGC 39BB at the presented cell voltage, and a very similar trend was observed at other cell voltages (Supplementary Figs. 2–3). The fact that the thicker GDLs must be compressed to a higher ratio for optimal operation (see Table 1), hence the catalyst layer is pressed to the membrane with higher force, however, can also be important.

**Table 1:** The most important technical parameters and the used notation of the GDLs (*: own data, **: ref. ^43^, ***: ref. ^44^).

Manufacturer	Type	Notation in the paper	Woven (W)/nonwoven (N)	Thickness/µm		PTFE in CFL/%	PTFE in MPL/%	Porosity %	Applied compression ratio/%
				Total	MPL				
Freudenberg	H23C6	FRG H23C6	N	250	37 ± 7^*^	n/a	n/a	61^**^	20
Freudenberg	H23C9	FRG H23C9	N	250	30 ± 7^*^	n/a	n/a	60^**^	10
Freudenberg	H14C9	FRG H14C9	N	180	30	n/a	n/a	n/a	17
Freudenberg	H23C2	FRG H23C2	N	255	43^**^	0	40	56 ± 3^**^	12
Freudenberg	H23I2	FRG H23I2	N	222	0	n/a	0	n/a	21
Sigracet	39BB	SGC 39BB	N	315	93 ± 18^*^	5	20-25	n/a	13
Sigracet	28BC	SGC 28BC	N	235	74 ± 15^*^	5	23	36-37	15
Sigracet	39BA	SGC 39BA	N	280	0	5	0	n/a	20
Sigracet	39AA	SGC 39AA	N	280	0	0	0	80	29
Elat	1400 W	LT 1400 W	W	454	99 ± 41^*^	n/a	n/a	63	35-45
Elat	2400 W	LT 2400 W	W	490	n/a	n/a	n/a	31	13
Toray	060 with MPL	TH60 MPL	N	250	93 ± 8^*^	8-9	33-35	78	10
Toray	TGP-H-060 10%	TH60 10%	N	190	0	10	0	78	8
Toray	TGP-H-060 30%	TH60 30%	N	190	0	30	0	78	8
Toray	TGP-H-060 50%	TH60 50%	N	190	0	50	0	78	8
CeTech	W1S1010	CT 1010	W	365	81^**^	n/a	n/a	54 ± 3^**^	32
CeTech	W1S1009	CT 1009	W	410	72^**^	n/a	30^***^	54^*^	33
AvCarb	MGL190 PTFE treated	AvCarb 10%	N	190	0	10	0	78	8
AvCarb	MGL190 PTFE treated	AvCarb 30%	N	190	0	30	0	78	8
AvCarb	MGL190 PTFE treated	AvCarb 50%	N	190	0	50	0	78	8

The structure of the CFL, whether it was woven or non-woven, had little-to-no effect on the rate of CO_2_RR (Fig. 2D). We also compared the different types of the CFL, such as the straight fibers, spaghetti-like fibers and the woven fabric structure (Supplementary Fig. 5). Even in this comparison, very small differences were found. The only notable difference was the slightly lower selectivity when using spaghetti fiber containing GDLs, and the observation, that these GDEs always seemed to be more wet when disassembling the cell after the experiments, as confirmed by their color difference as compared to unused samples. On the contrary, droplets of water could be easily removed from the back of the used straight fiber based GDEs (e.g., SGC 39BB).

**Fig. 3:**
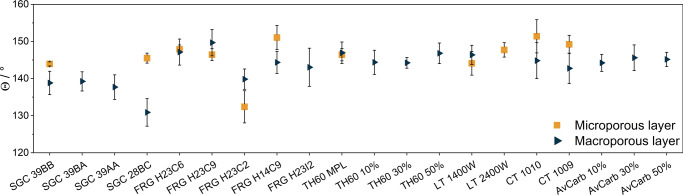
Wetting properties of different commercially available carbon gas diffusion layers. Contact angles measured with 10 µl 0.1 M CsOH solution. Each data point and standard deviation is calculated from results measured for at least 5 separate liquid droplets. The results for the microporous layer are denoted with yellow squares, for the macroporous layer with blue triangles.

To further understand the differences observed among the behavior of the various GDLs, we characterized their wetting properties (Fig. 3) and microstructure before the electrochemical measurements. We found that only the contact angle of a GDL without PTFE content (FRG H23C2) was <140° (131°), while a value between 140–150° was measured for all other GDLs, indicating a hydrophobic surface. As for the support layer (CFL) side, the deviation was larger, which could be attributed to their different PTFE content. An important conclusion from these measurements is that the highest contact angle and the best performing GDLs do not overlap (Supplementary Fig. 6). This means that a high contact angle does not necessarily ensure outstanding electrochemical performance. The reason behind this is most probably that high amounts of PTFE must be incorporated in the GDL to achieve very high contact angles. This in turn might partly block the pores in the GDL, decreasing the cell performance by altering the gas supply to the cathode catalyst layer. Finally, we note that these values were determined on unused GDLs, before catalysts deposition and electrolysis. Contact angles can change notably during operation, as it was witnessed in earlier studies^39^.

**Fig. 4:**
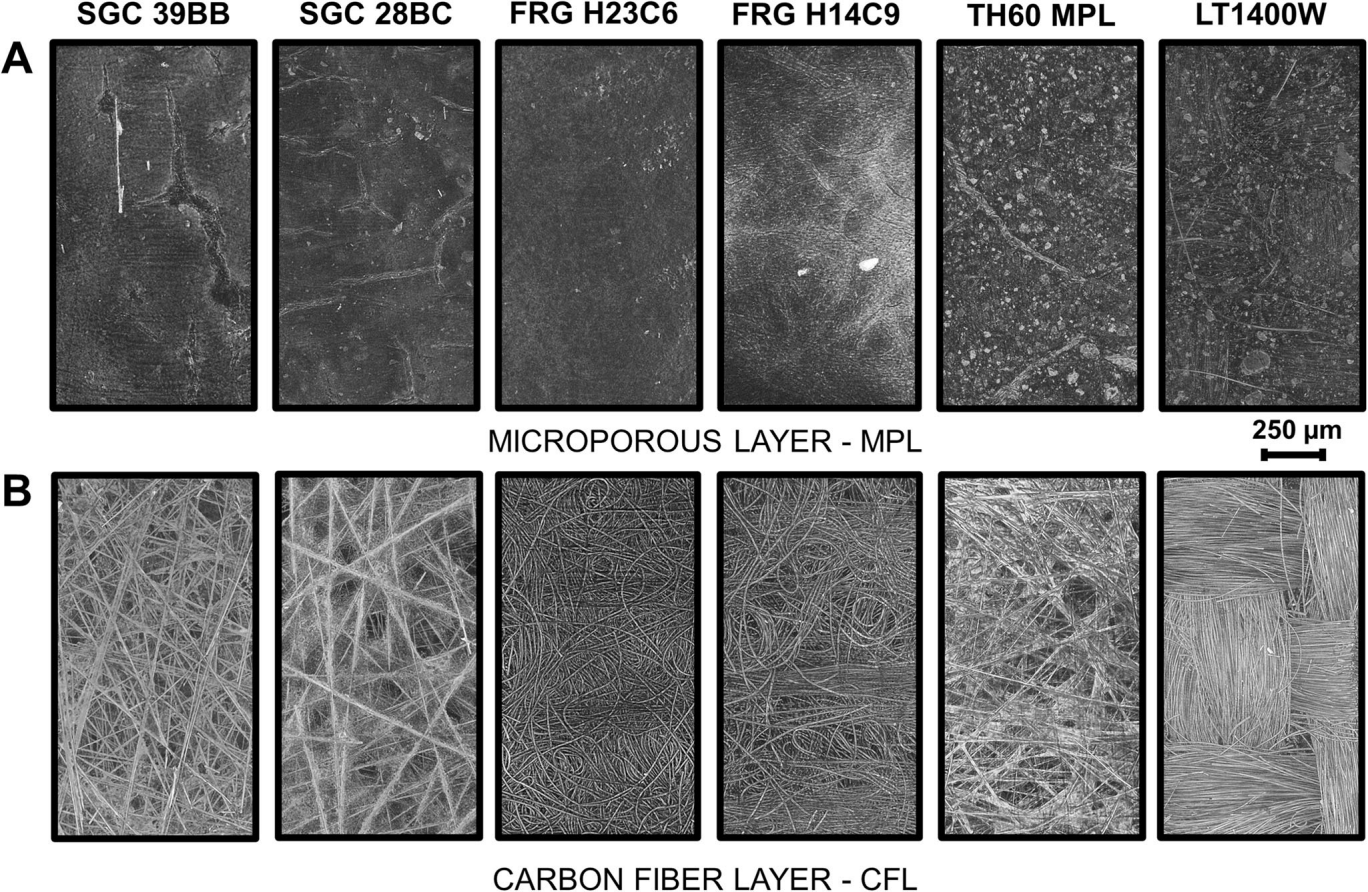
Structure of different commercially available carbon gas diffusion layers. X-ray computed tomography of selected gas diffusion layers: (**A**) shows the side covered by the microporous layer, while (**B**) shows the other side.

X-ray computed tomography (micro-CT) was employed to study morphological and surface roughness differences among the six best performing GDLs (Fig. 4). The images of the two SGC GDLs show that these were assembled of straight carbon fibers, and that the gap among the fibers is partly filled with the PTFE binder. This was more emphasized for the thinner GDL (SGC 28BC), showing a larger PTFE content for this sample. This phenomenon is also visible on the SEM images of the CFL structures (Supplementary Fig. 5).

**Fig. 5:**
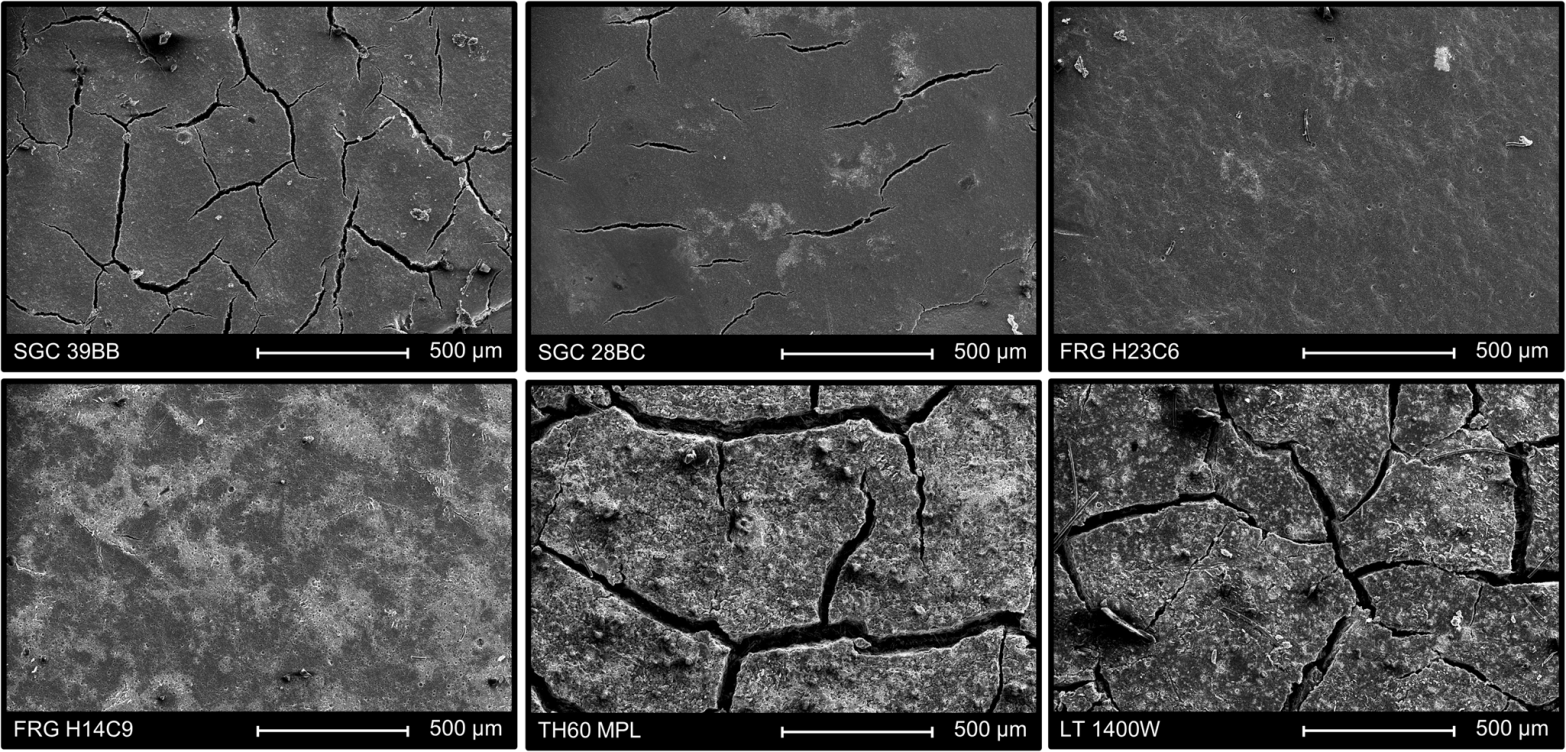
Morphology of the microporous layer of different carbon gas diffusion layers. SEM images of the six selected gas diffusion layers’ microporous layer side.

The MPL thickness is clearly dissimilar for the different GDLs (Supplementary Fig. 7). The thinnest MPL was found for the two FRG GDLs, which were about 28–34 µm. As the other extreme, the average thickness of the TH60 GDLs MPL was 93 ± 15 µm (Supplementary Table 1). In case of the FRG H14C9 and FRG H23C6 GDLs, the surface of the MPL was homogeneous and coherent, but for the other GDLs it contained a large amount of cracks (Fig. 5, Supplementary Fig. 8 and Supplementary Table 2).

One of the main differences noticed from the SEM images for the GDLs, was in the number and size of the cracks on the surface of the MPL. The TH60 MPL GDL contained the most and largest cracks (cracks covered ca. 12% of the selected area), and we observed several small cracks on the surface of the SGC 28BC GDLs (only 2% was covered with cracks). Interestingly, we found that GDLs with more cracks, such as the SGC 39BB, TH60 MPL and the LT 1400 W performed better in CO_2_RR (in terms of CO partial current density at a given cell voltage, Supplementary Fig. 9).

The width of cracks was on average 40 µm for TH60 MPL and 10 µm for SGC 28BC. These cracks could play an important role in the water and gas managements, as indicated by recent reports^33,38^. We refer to similar studies on fuel cells, where the important role of cracks in water management and mechanical stability of the GDLs was shown^40,41^. In zero-gap CO_2_ electrolyzer cells, water is supplied to the cathode from two sources—from the humidified gas stream and from the diffusion of water from the electrolyte solution through the membrane. This latter occurs directly at the cathode catalyst surface, which might decrease the reaction rate and selectivity by blocking part of the electrochemically active surface area and increasing the diffusion length of CO_2_. In line with earlier results, therefore, we think that the cracks in the GDE are beneficial in this case. This conclusion, however, is not a general one, as water management very much depends on the structure of the electrolyzer cell and on the electrolysis conditions.

We further correlated our electrochemical measurements with reported capillary breakthrough pressure values^42^, but we found no direct correlation between these and the measured CO formation rates. What is clear, that the gas breakthrough pressure is minimal for all the tested carbon papers. The liquid breakthrough pressure is highest for the crack-free Freudenberg carbon papers, but it is very similar for all the GDLs with cracked MPL (Sigracet, Toray, ELAT). This, however, does not correlate with the electrochemical measurements, as both cracked and non-cracked GDLs were among the best performing substrates.

**Fig. 6:**
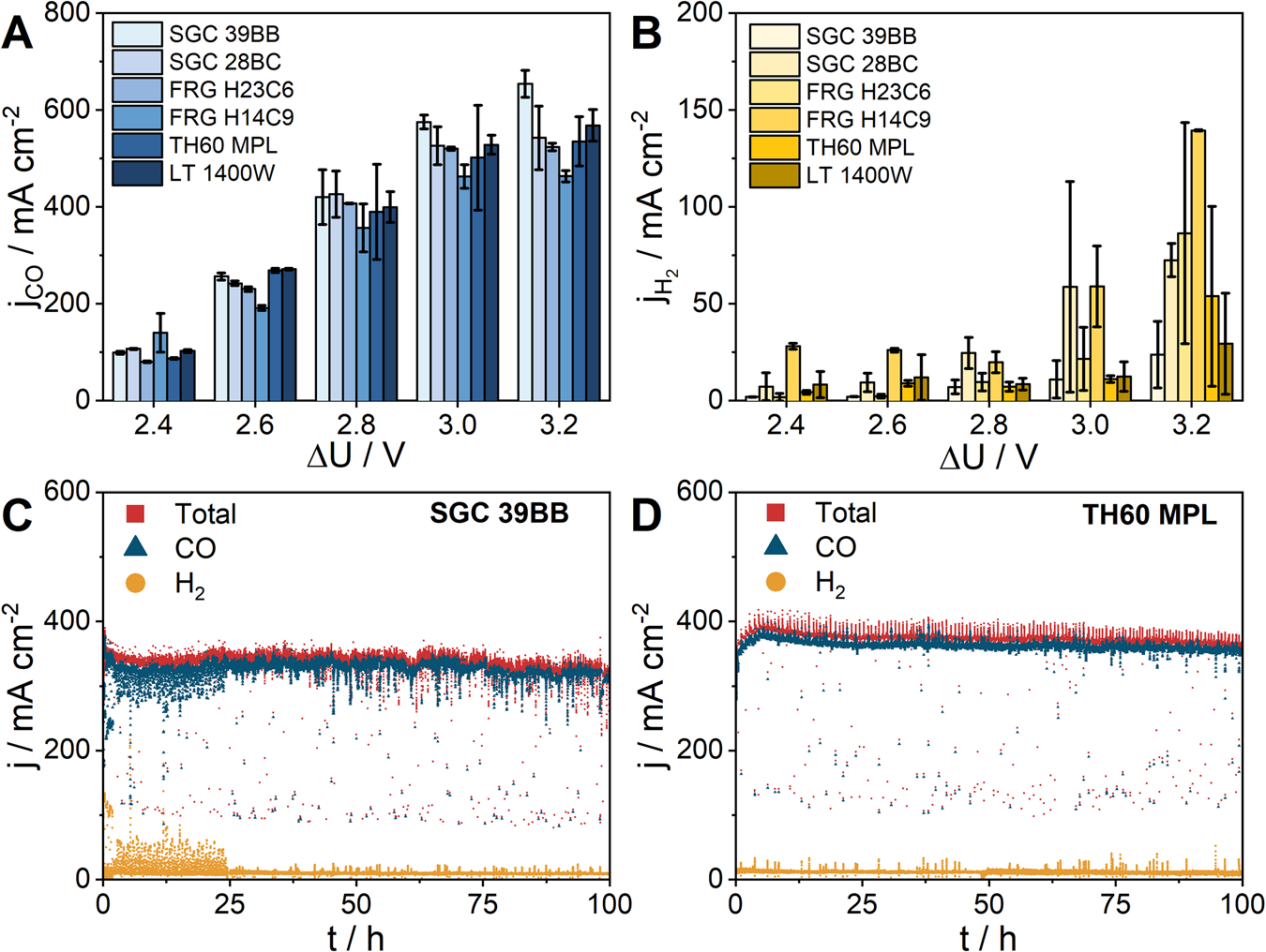
The performance of a CO_2_ electrolyzer cells with different gas diffusion layers. **A**, **B** Partial current densities for CO (shades of blue) and H_2_ (shades of yellow) formation during CO_2_ reduction reaction at different cell voltages using a 0.1 M CsOH anolyte and **C**, **D** 100-hours long electrolysis at ΔU = 2.8 V in 0.05 M CsOH anolyte. Total current densities are shown with red squares, while the partial CO and H_2_ current densities are represented with blue triangles and yellow circles, respectively. The CO_2_ feed rate was 12.5 cm^3^ cm^−2^ min^−1^, and the electrolyzer cell temperature was 60 °C for all measurements. The error bars in **A** and **B** represent the deviations from two consecutive analyses during two separate measurements.

The six best performing GDLs were further studied at five different cell voltages (Fig. 6A-B). These measurements were repeated on another set of GDLs from the same batch. Consequently, the error bars in Fig. 6A-B represent the deviations of four data points acquired during two different measurements. All the selected GDLs showed above 200 mA cm^−2^ partial CO current density already at 2.6 V cell voltage. At 3.0 V cell voltage, the GDLs performed almost identically, with a CO partial current density of 460-580 mA cm^−2^. In the case of the thinner GDLs (SGC 28BC and FRG H14C9), the HER partial current density increased notably already at 3.0 V, it reached almost 50 mA cm^−2^. When we employed 3.2 V cell voltage, four GDLs (SGC 39BB, SGC 28BC, TH60 MPL and LT 1400 W) achieved >530 mA cm^−2^ partial CO current density, and the CO/H_2_ ratio was still over 10 for SGC 39BB, TH60 MPL and LT 1400 W, indicating selective operation.

Long-term electrolysis experiments (100 h) were performed with two GDLs, the SGC 39BB (Fig. 6C) and the TH60 MPL (Fig. 6D). Throughout the long-term electrolysis, a stable total current density and CO formation selectivity was measured in both cases: a degradation rate of dj_CO_/dt ≈ 140 µA cm^−2^ h^−1^ was determined in both cases for the last 3 days of the measurements (the first 24 h was considered as a transient period, as a current increase occurred with the TH60 MPL GDL, that would mathematically result in a negative degradation rate). Importantly, the measurements were continuous, and we did not wash the cathode current collector with water or any other solution. After disassembling the electrolyzer cells, no physical precipitate formation was witnessed. We mention that similar experiments were performed with the thinner SGC GDL, but in this case we observed a more rapid performance decay (ca. 340 µA cm^−2^ h^−1^ for the last 3 days of the experiment, Supplementary Fig. 10). We note that this conclusion is only valid under these specific experimental conditions. To fully address the role of the GDL on the cell performance decay, however, the experimental conditions (e.g., temperature, humidity, anolyte concentration, gas flow rate) should be optimized for each GDL separately in sufficiently long experiments (e.g., 100 h). These experiments are ongoing in our laboratory but point beyond the scope of the current study.

## Conclusions

In this study, we tested 20 different commercially available GDLs ordered from six manufacturers for the electrochemical reduction of CO_2_. We investigated the influence of the MPL, the PTFE content, the thickness, and the structure of the GDLs under identical, highly controlled experimental conditions.

We have demonstrated that in the absence of an MPL, the HER was the favored cathode process, irrespective of other parameters, such as the PTFE content of the GDL. The thickness of the GDLs does not exert a significant effect in the short-term electrolysis experiments, although a slight increase in CO selectivity was witnessed with the increasing thickness. During the long-term measurements (100 h continuous electrolysis) we found that using a thicker GDL ensures more stable reaction rate and CO selectivity. The effect of the cracks in the MPL of GDLs is not yet fully clear, but under the applied experimental conditions and electrolyzer cell configuration (zero-gap), GDLs with more crack led to the best results. By further investigating the six best performing GDLs we showed that CO formation current densities above 200 mA cm^−2^ can be routinely achieved already at 2.6 V.

## Methods

**Fig. 7:**
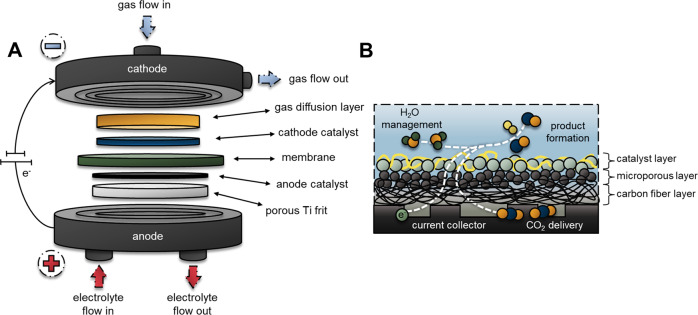
Illustration of the applied electrolyzer cell and the processes occurring at the cathode. **A** Schematic illustration of the zero-gap electrolyzer cell. **B** Schematic illustration of the structure of the gas diffusion electrode structure.

In our work, we employed a zero-gap electrolyzer cell (*A* = 8 cm^2^), where the catalyst coated electrodes are separated by only a Grade-T Sustainion^®^ X37-50 anion exchange membrane (AEM). Schematic illustration of our cell design is shown in Fig. 7A. In this cell configuration, both electrodes are directly pressed to the AEM, with the catalyst layers facing the membrane. The cells were designed and assembled in a way to compress the cathode GDE to a precisely controlled thickness. This compression guarantees the electronic contact between the components without damaging the structure of the GDL, and forces the CO_2_ gas in the porous GDE. In our custom-designed electrolyzer cell the spacing of the cathode GDE is set by a PTFE gasket. To vary the compression ratio of the cathode GDE, we used different thickness PTFE gaskets to control the spacing for the cathode. This compression ratio was optimized for each GDLs separately, starting from a cathode spacing matching the total thickness of the GDE, and perform a set of experiments in which this spacing is gradually lowered (see Supplementary Fig. 11 for exemplary measurements, and Table 1 for the optimized compression ratio of the different GDLs). Here we only present results gathered at the optimal values.

Ag nanoparticle coated GDL-based GDEs were used as cathode, and Ir coated 1 mm thick porous Titanium frit was employed as anode. For all experiments, we used the same 1.0 mg cm^−2^ catalyst loading for both electrodes. During our experiments, the temperature of the electrolyzer cell was 60 °C, the electrolyte solution (0.1 M CsOH for the short measurements and 0.05 M CsOH for the long-term electrolysis) was recirculated continuously through the anode side, while humidified CO_2_ was fed to the cathode side of the cell with 12.5 cm^3^ cm^−2^ min^−1^ flow rate. The structure and morphology of the GDLs (and GDEs) were characterized with SEM, micro-CT and contact angle measurements. Further information on electrode preparation and characterization is provided in the Supplementary Methods.

Throughout our study, we focused on the effect of the structure of GDLs on CO_2_RR (Fig. 7B). The thickness of GDLs varied between 150 and 500 µm. Beyond the GDLs most frequently applied in CO_2_RR studies, we chose GDLs that can be compared to at least one of these, as they differ in only one key descriptor (Table 1). We tested the influence of the MPL, the PTFE content, the thickness, and the structure of the GDLs.

## Supplementary information

Supplementary Information

## Data Availability

The data that support the findings of this study are available from the corresponding author upon reasonable request.
