# Supplementary material for: Systematic screening of gas diffusion layers for high performance CO2 electrolysis
Source: Commun Chem. 2023 Feb 24;6:41. doi: 10.1038/s42004-023-00836-2 (PMC9958001; doi:10.1038/s42004-023-00836-2)
Supplement: Supplementary file 1 — Supplementary Information [file 42004_2023_836_MOESM1_ESM.pdf]

# – Supplementary Information –

Systematic screening of gas diffusion layers for high performance CO_2 electrolysis

Angelika Anita Samu[1,2], Imre Szenti[3], Ákos Kukovecz[3], Balázs Endrődi[1*] and Csaba Janáky[1,2*]

[1]Department of Physical Chemistry and Materials Science, University of Szeged, Rerrich Square 1, Szeged, H-6720, Hungary

[2]eChemicles Zrt, Alsó Kikötő sor 11, Szeged, H-6726, Hungary

[3]Department of Applied and Environmental Chemistry, University of Szeged, Rerrich Square 1, Szeged, H-6720, Hungary

Supplementary Methods

Experimental Methods

Preparation of Electrodes

We dispersed 25 mg cm^{-3} Ag nanoparticles (d_{avg} < 100 nm, Sigma-Aldrich) in a 1:1 isopropanol-water solvent mixture to prepare the cathode catalyst ink. This dispersion ink also contained 15 wt% Sustainion® XA-9 alkaline ionomer (Dioxide Materials) as a catalyst binder. The anode catalyst ink contained 20 mg cm^{-3} Ir nanoparticles (d = 4-6 nm, Fuel-Cell Store) in an identical solvent mixture with identical ionomer content. These dispersions were homogenized for at least 20 minutes in an ultrasonic bath (Elmasonic P 30 H) prior to electrode preparation. We also used a high-power immersion sonotrode (Hielscher UP200ST) to fully disperse the aggregated Ag nanoparticles for 3 minutes and the power of 200 Watt. The cathode and anode catalyst layers were formed by spray-coating, using a hand-held airbrush (Alder AD-320). The dispersions were spray-coated on samples preheated on a hotplate at 100 °C, at a constant flow rate. The only difference in the spray coating method was the supporting material, because for the anode catalyst we used a 1 mm thick, porous Ti frit, while carbon-based gas diffusion layers (GDLs) were used as cathode catalyst supports (from FuelCellStore and FuelCellEarth). The cathode and the anode catalyst loadings were 1.0 ± 0.1 mg cm^{-2}. Before inserting the cathode gas diffusion electrodes (GDEs) in the electrolyzer cell, these were immersed in a 0.1 M CsOH (Sigma-Aldrich) solution for at least 15 minutes, to convert the applied ionomer to hydroxide form (the catalyst layer facing the liquid surface). After this preparation, we cut into shape with a hollow punch. In all cases the active surface of the cathode electrode was an 8 cm^2 circle. We collected all relevant parameters of the studied gas diffusion layers in the manuscript (Table 1). For the Sigracet GDL's, the nomenclature describes the specifications. The "SGC AA" is a simple substrate with no added PTFE or MPL, while the "SGC BA" is a hydrophobized substrate with a 5 wt% PTFE treatment with no MPL. The "SGC BB/BC" is a hydrophobized substrate (5 wt% PTFE) with MPL on one side.

Electrolyzer cell assembly and test framework

A custom-designed direct gas feed zero-gap electrolyzer cell was used for all experiments. The cell consists of two catalyst-coated electrodes, which were separated with only a 60 μm thick PTFE reinforced anion exchange membrane (Sustainion® X37-50 grade T, Dioxide Materials). Before use, the membrane was activated by immersing it in a 1.0 M CsOH solution for at least 24 hours. After activation, it was cut into shape using a sharp surgical blade and washed with deionized (DI) water before inserting it into the electrolyzer cell. The electrodes were placed in the cell with the catalyst layers facing the membrane (hence each other as well). Six bolt screws were used to assemble the cell, with a final, gradually applied torque of 3 Nm. The anolyte (CsOH solution) was circulated with a peristaltic pump through the anode current collector at a flow rate of 80 $cm^3 min^{-1}$. Through the cathode current collector, the humidified (at T = 60 °C) CO_2 stream was supplied at a flow rate of 12.5 $cm^3 cm^{-2} min^{-1}$, controlled with a Bronkhorst MASS-STREAM D-6321 type mass-flow controller. The short-term (ca. 1 hour at each applied cell voltage) electrochemical measurements presented in this paper were performed using a Biologic VMP- 300 type instrument, equipped with high current (up to 10 A) booster. A Rohde & Schwarz HMP4040 power supply was applied for the long-term operations. The composition of the cathode product stream during the short electrochemical measurements was analyzed with a Shimadzu Nexis-GC-2030 type instrument, equipped with a barrier discharge ionization (BID) detector. A Restek ShinCarbon ST column was employed for the separation, with 6.0 grade He carrier gas. For the long-term operations we used an online infrared-thermal conductivity gas analyzer (Gasboard-3100, customized for CO_2-CO-H_2 mixtures, Hubei Cubic-Ruiyi), which allowed a real-time determination of the product composition for extended measurement times (100 hours in our case). A sample protocol of our electrochemical measurement is shown in Supplementary Figure 1.

A sample protocol of our electrochemical measurement

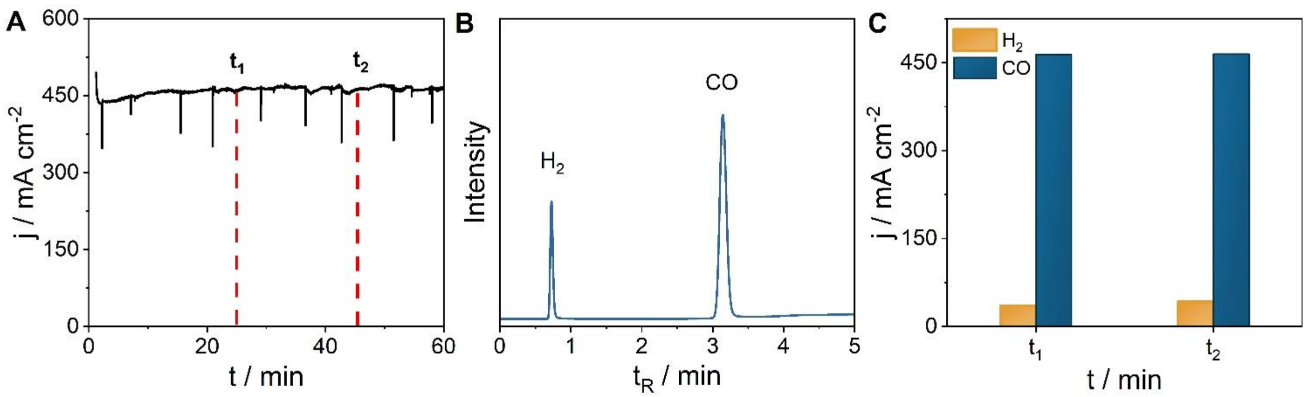

Supplementary Figure 1. (A) Chronoamperometric curve recorded during CO_2RR at ΔU = 2.6 V, applying 0.1 M CsOH electrolyte solution. The CO_2 feed rate was 12.5 cm^3 cm^{-2} min^{-1}, and the electrolyzer cell temperature was T = 60 °C. The two red lines denote the analysis times (t_1, t_2) of the gas samples. (B) Chromatogram of the analyzed gas samples during the measurement. (C) Partial current densities for CO and H_2 formation.

Characterization methods

X-ray micro-computed tomography analysis

The GDLs were scanned using a Bruker Skyscan 2211 nano-CT cone-beam scanner (Skyscan, Bruker, Belgium) with X-ray source settings of 50 kV source voltage, 600 μA source current and 350 ms exposure time in micro-focus mode The applied detector was a 11 Mp active pixels CCD detector at 700 nm pixel resolution. 1940 X-ray projections were collected through a 180° rotation of the sample with 0.1° angular step size in around 4 hours.

The acquired images were reconstructed by volumetric NRecon Reconstruction Software (Skyscan, Bruker, Belgium). During reconstruction typically occurring artifacts, such as ring artifact and beam hardening artifacts were corrected. The 3D models of the samples were created using CTVox 3D Micro-CT Volume Rendering software (Skyscan, Bruker, Belgium).

Scanning electron microscope analysis

To characterize the surface morphologies of the GDLs we used a Thermo Fisher Scientific Scanning Electron Microscope (SEM) using Everhart–Thornley detector (ETD). The microscope was operated at 25 pA current and 10 kV acceleration voltage.

Contact angle measurements

The static contact angles were measured using an EasyDrop (Krüss GmbH, Hamburg, Germany) type instrument. The experiments were analyzed with a DSA100 software. The contact angles were measured at both the micro- and macroporous sides of the GDLs. A drop (10 μl) of 0.1 M CsOH solution was formed on the surface with the use of a steel syringe of 0.5 mm diameter. Using the CCD camera of the goniometer, we analyzed the photographs of the droplets. This experiment was repeated 5 times. The dynamic wetting of GDLs was also measured but in this case, we used a PTFE syringe of 0.5 mm diameter. The measured advancing and receding contact angles were plotted.

Chronoamperometric measurements performed at different cell voltages

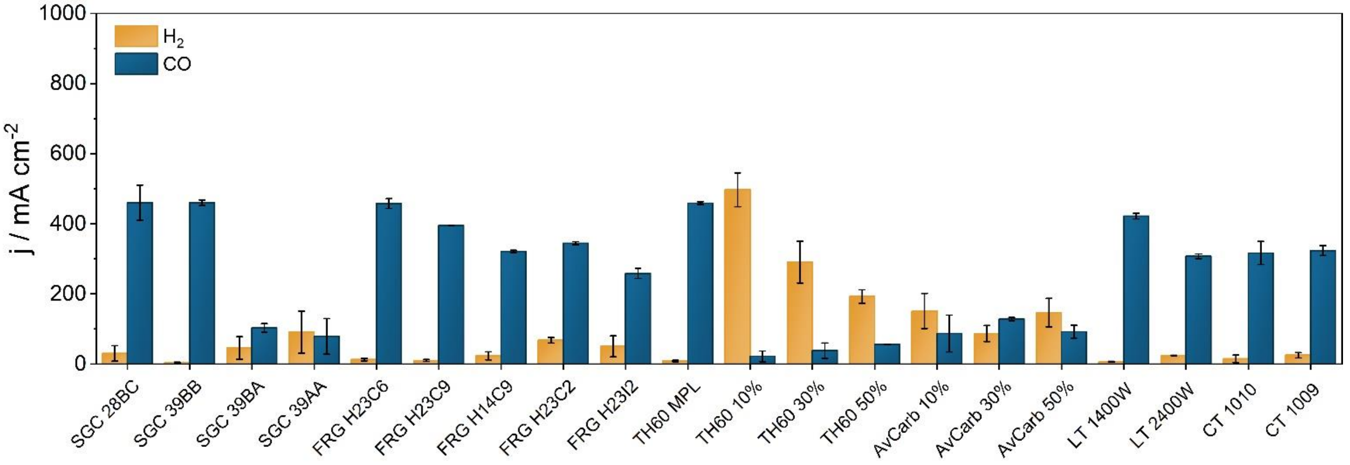

Supplementary Figure 2. Partial current densities for CO and H_2 production during one-hour long chronoamperometric CO_2RR measurements at ΔU = 2.8 V, applying different GDL based cathode GDEs with Ag catalyst. The electrolyte was 0.1 M CsOH solution, humidified CO_2 was fed to the cathode at a flow rate of 12.5 $cm^3\ cm^{-2}\ min^{-1}$ and the temperature of the electrolyzer was 60 °C. The error bars represent the deviations of two consecutive analyses during the same measurements.

Chronoamperometric measurements performed at different cell voltages

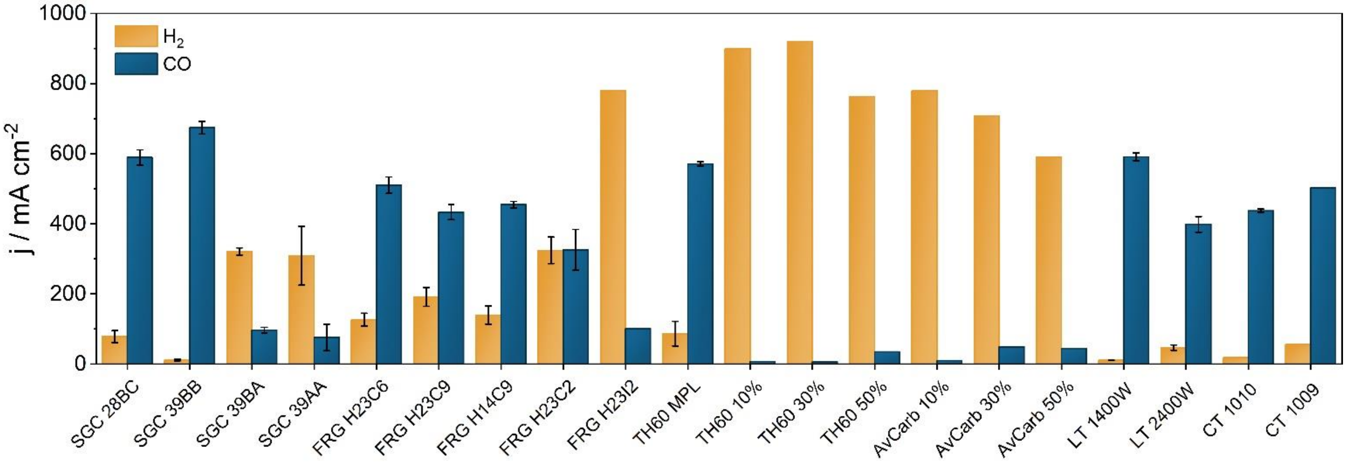

Supplementary Figure 3. Partial current density for CO and H_2 production during one-hour long chronoamperometric CO_2RR measurements at ΔU = 3.2 V, applying different GDL based cathode GDEs with Ag catalyst. The electrolyte was 0.1 M CsOH solution, humidified CO_2 was fed to the cathode at a flow rate of 12.5 cm^3 cm^{-2} min^{-1} and the temperature of the electrolyzer was 60 °C. The error bars represent the deviations of two consecutive analyses during the same measurements.

Impedance spectroscopic investigation of the effect of PTFE content in the GDL

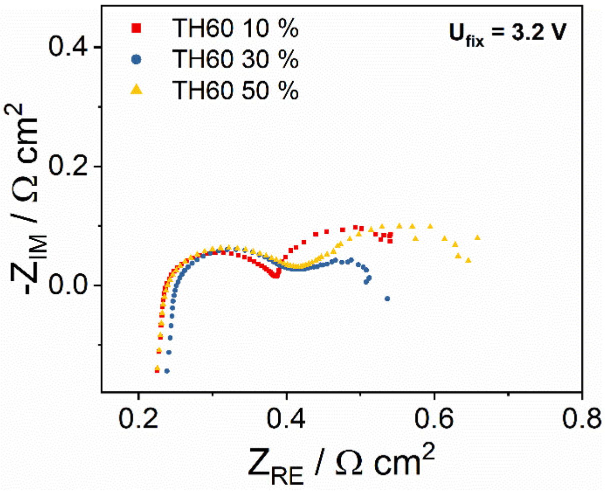

Supplementary Figure 4. EIS spectra of cells assembled and operated identically, with GDLs only differing in their PTFE content. The electrolyte was 0.1 M CsOH solution, humidified CO_2 was fed to the cathode at a flow rate of 12.5 cm^3 cm^{-2} min^{-1} and the temperature of the electrolyzer cell was 60 °C.

The high frequency intercepts (HFR) of the arcs are within experimental error for the studied GDLs. This suggests that the contribution of the GDL to the total cell resistance is negligible. This is further confirmed by the area specific resistivity values, provided by the producers; as an example, this value is < 10 mΩ cm^2 for the used Sigracet substrates. As for the membrane, we determined its carbonate conductivity to be around 15 mS/cm under the applied conditions. From this, its resistivity is 66.7 Ω cm. Area specific resistance (ASR) is defined as $ASR = R \times A$, while the resistivity is $\kappa = R \times \frac{A}{l}$, where A is the surface area, while l is the thickness of the membrane (50-60 µm in our case). From this, the ASR is about $\kappa \times l = 0.3 - 0.4\Omega\ \mathrm{cm}^2$, that is very close to the measured HFR.

SEM images of the six selected GDL's CFL side

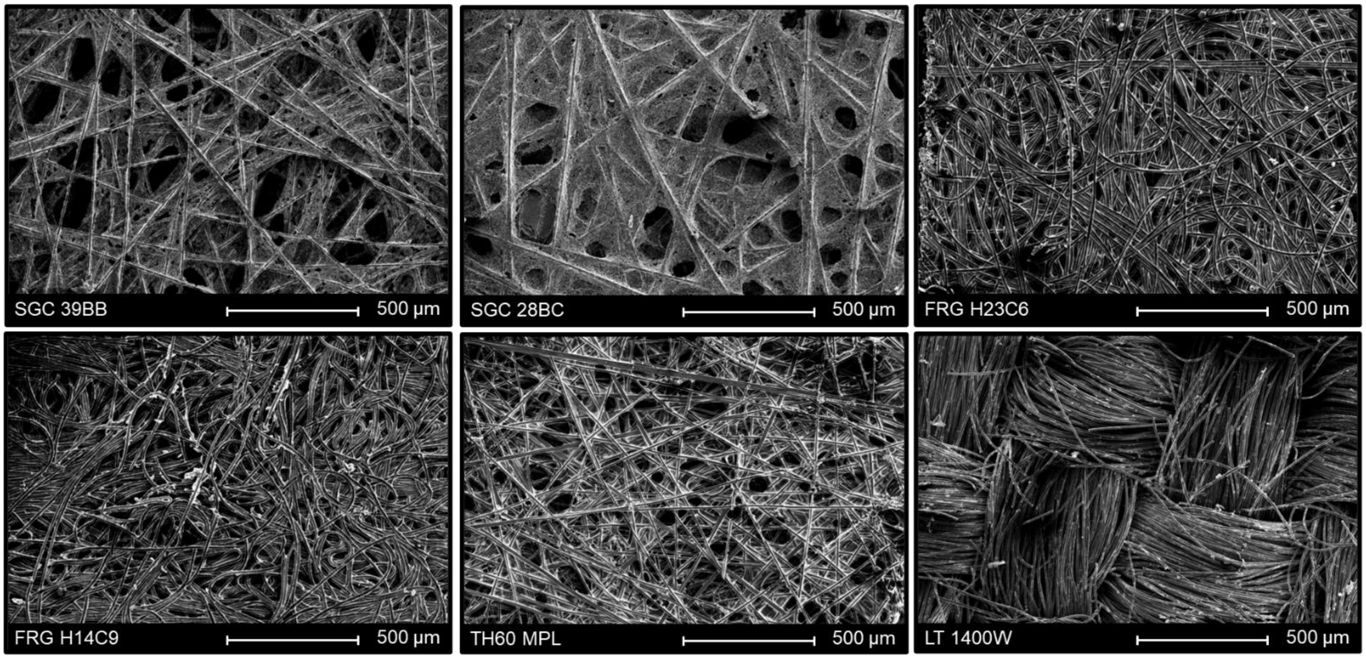

Supplementary Figure 5. SEM images of six selected (SGC 39BB, SGC 28BC, FRG H23C6, FRG H14C9, TH60 MPL, LT 1400W) GDLs' CFL side.

Maximum partial CO current densities against the contact angles at different cell voltages

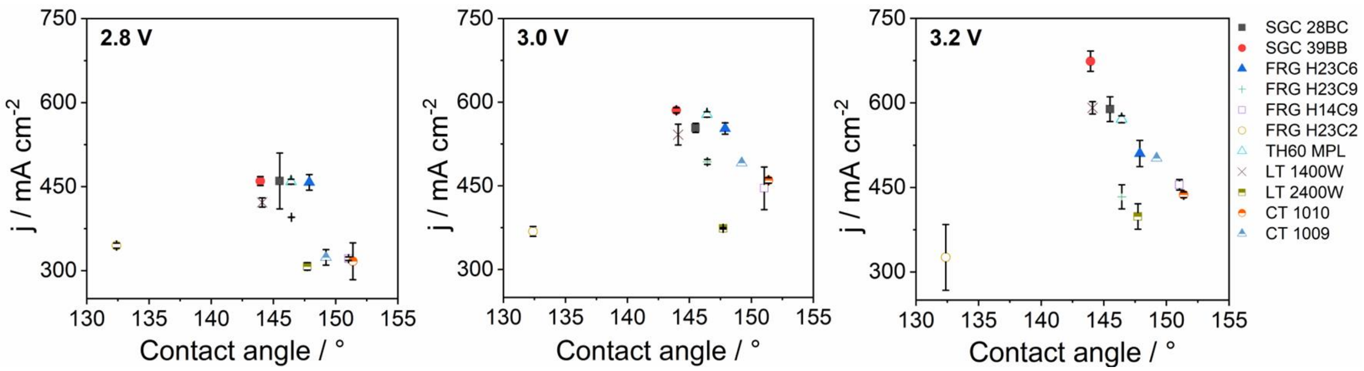

Supplementary Figure 6. Maximum partial CO current densities plotted against the static contact angles at different cell voltages, applying different GDL based cathode GDEs. For the wetting contact angles, we measured a drop (10 µl) of 0.1 M CsOH solution on the surface.

Cross-sectional microCT images of the six selected GDLs

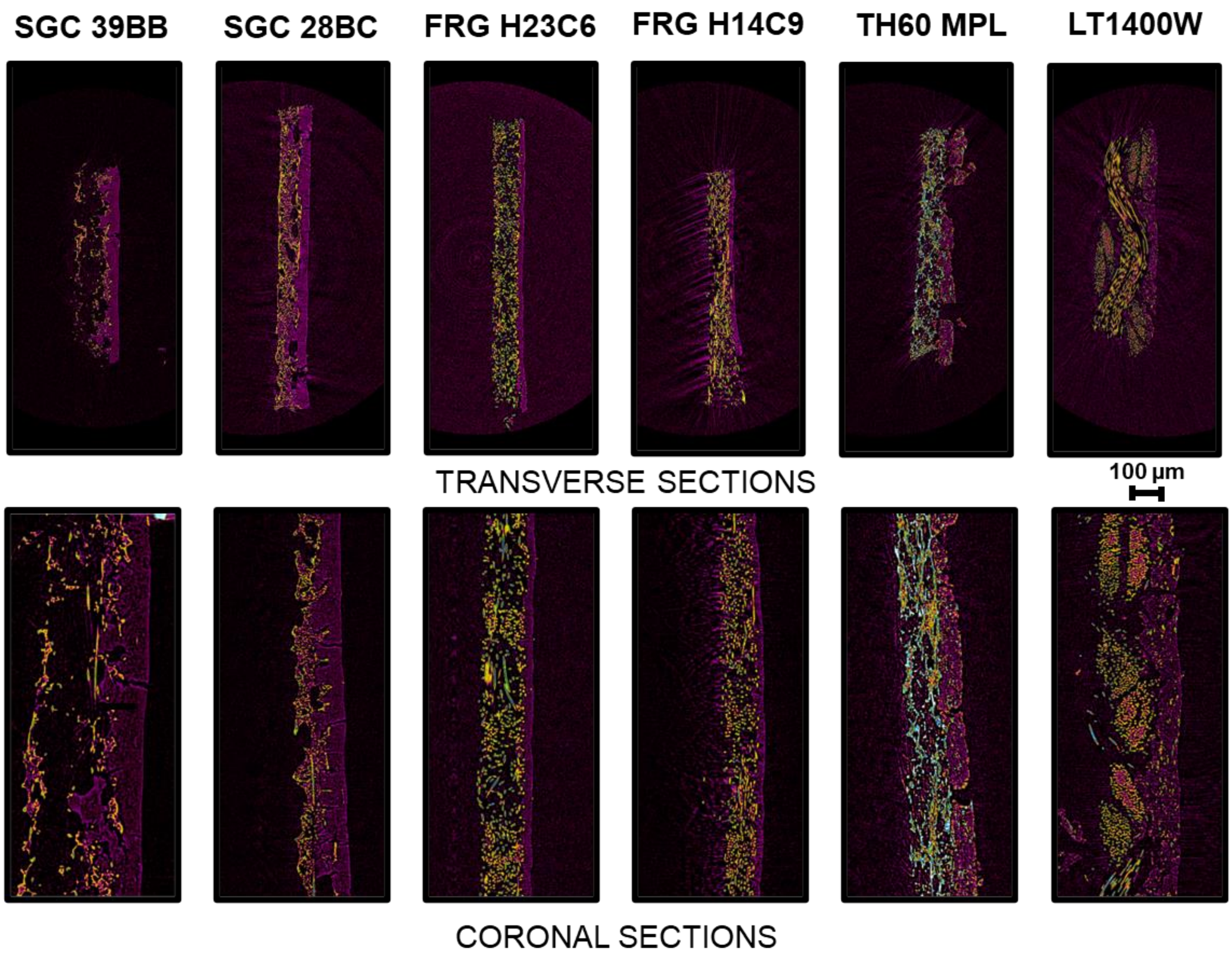

Supplementary Figure 7. Cross-sectional microCT images of the six selected GDLs.

Supplementary Table 1. The minimum, maximum and the average thicknesses of the MPLs of the GDLs.

Gas Diffusion Layer	Minimum thickness of MPL / µm	Maximum thickness of MPL / µm	Average thickness of MPL / µm
SGC 39BB	73	131	93 ± 18
SGC 28BC	53	108	74 ± 15
FRG H23C6	25	49	37 ± 7
FRG H14C9	20	42	30 ± 7
TH60 MPL	81	105	93 ± 8
LT 1400W	53	178	99 ± 41

SEM analysis of the surface cracking of different GDLs

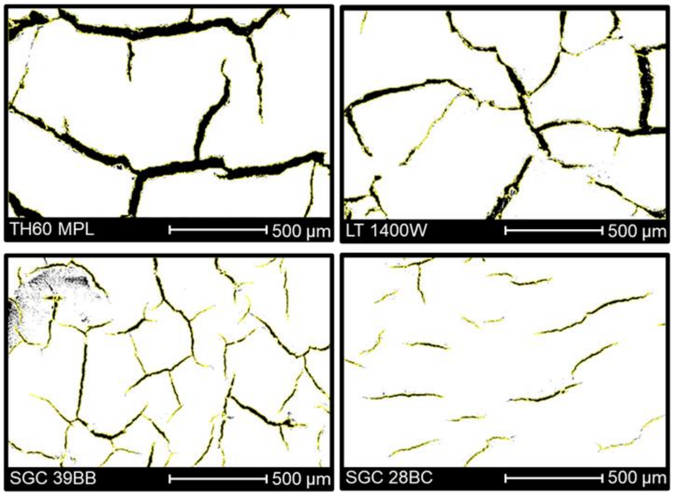

Supplementary Figure 8. SEM images of the selected GDLs after black-and-white transformation of the images shown in Figure 6. in the main text.

Supplementary Table 2. The average, minimum, maximum width of the cracks on the surfaces of the GDLs.

Gas diffusion layer	Average width of cracks / µm	Minimum width of cracks / µm	Maximum width of cracks / µm	Coverage of the surface / %
SGC 39BB	15 ± 6	5	28	5
SGC 28BC	11 ± 3	5	18	2
FRG H23C6	0	0	0	0
FRG H14C9	0	0	0	0
TH60 MPL	41 ± 14	16	65	12
LT 1400W	31 ± 12	11	60	8

Maximum partial CO current densities against the crack coverage of the surface at different cell voltages

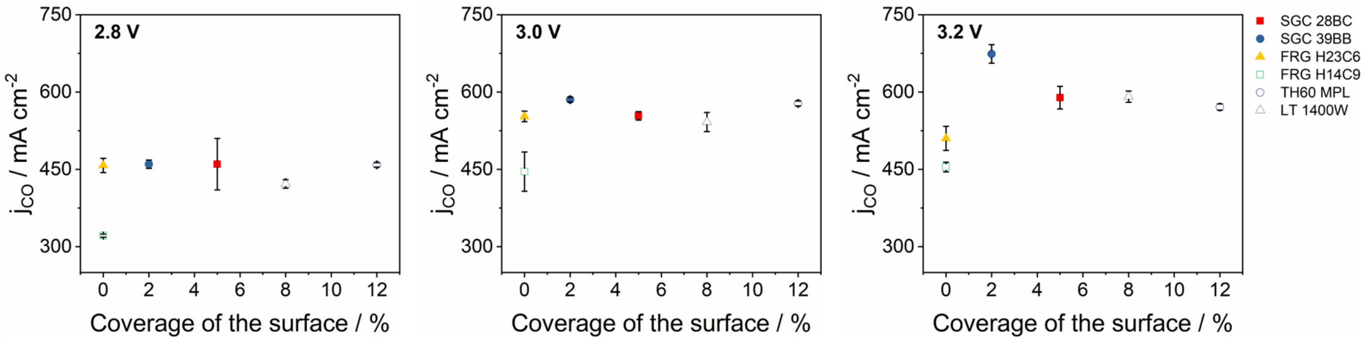

Supplementary Figure 9. Maximum partial CO current densities plotted against the crack coverage of the surface at different cell voltages, applying different GDL based cathode GDEs.

Long-term CO_2RR measurements at fixed cell voltage

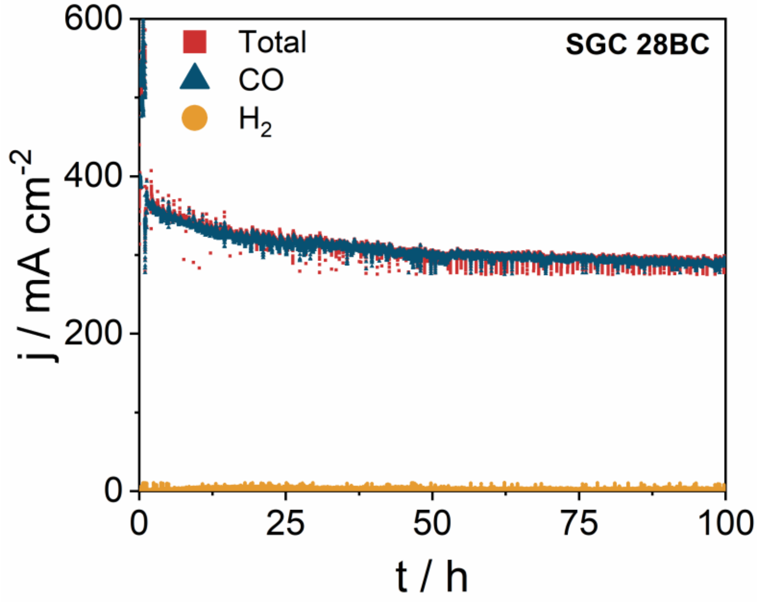

Supplementary Figure 10. Partial current densities for CO and H_2 production during 100-hours long chronoamperometric CO_2RR measurement at ΔU = 2.8 V, with 0.05 M CsOH electrolyte solution, applying a SGC 28BC GDL based Ag GDE. The CO_2 feed rate was 12.5 cm^3 cm^{-2} min^{-1}, and the cell temperature was 60 °C.

Effect of the GDE compression ratio on the partial current densities

The ratio of GDE compression was calculated from the original total thickness of the GDE (d_{total}) and the thickness set (d_{set}) by the spacing element, according to the following formula:

$$Ratio\ of\ compression = \frac{d_{total} - d_{set}}{d_{total}}$$

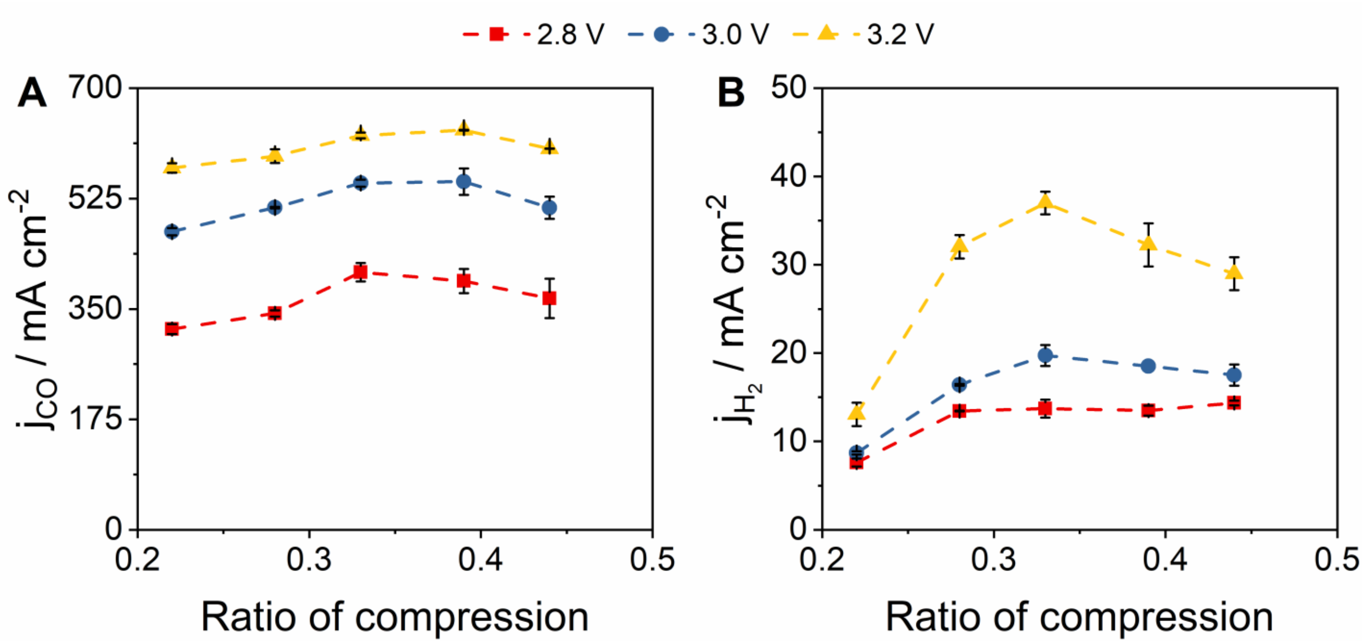

Supplementary Figure 11. Partial current densities for (A) CO and (B) H_2 production during one-hour long chronoamperometric CO_2RR measurements at different cell voltages (2.8, 3.0 and 3.2 V), applying different ratio of compression of the LT 1400W GDL based Ag GDE. The electrolyte solution was 0.1 M CsOH, humidified CO_2 was fed to the cathode at a flow rate of 12.5 cm^3 cm^{-2} min^{-1} and the temperature of the electrolyzer was 60 °C. The error bars represent the deviations of two consecutive analyses during the same measurements and the dashed lines in the figure are guide for the eye.
